# Diffuse Pigment Release in a Patient Undergoing Tumor-Infiltrating Lymphocyte Immunotherapy for Acral Malignant Melanoma

**DOI:** 10.18502/jovr.v18i3.13783

**Published:** 2023-07-28

**Authors:** Madison Kerley, Niloofar Piri, Aparna Ramasubramanian

**Affiliations:** ^1^Department of Ophthalmology and Visual Sciences, University of Louisville School of Medicine, Louisville, KY; ^2^Department of Ophthalmology, Saint Louis University, St. Louis, MO; ^3^Department of Ophthalmology, Phoenix Children's Hospital, Phoenix, AZ

##  PRESENTATION

Although acral malignant melanoma accounts for 2% of melanoma diagnoses, it has the lowest five- and ten-year survival rates of melanoma subtypes.^[1]^ Its general presentation is a rapidly growing pigmented region on the soles of feet or the palms of hands.^[1]^ Treatment includes lesion excision, often with lymph node biopsy, molecularly targeted therapy, chemotherapy, and immunotherapy.^[2]^

Tumor-infiltrating lymphocyte immunotherapy (TIL) is an adoptive cell transfer variant where tumor-infiltrating lymphocytes extracted from a metastatic melanoma tumor are cultured with IL-2, which serves to activate T-cells. After extraction, the patient is given non-myeloablative lympho-depleting chemotherapy. Chemotherapy allows for rapid growth of the IL-2 cultured lymphocytes when they are re-introduced to the body.^[3]^ The lymphocytes recognize tumor-related antigens, directly attacking cancer cells.

Although TIL has proven to be a powerful treatment for metastatic melanoma, it can lead to melanocyte-reactive T cells in other melanocyte containing regions.^[3]^ Herein, we present a unique case of acral metastatic melanoma, treated by adoptive cell transfer leading to asymptomatic intraocular pigmentary changes.

**Figure 1:**
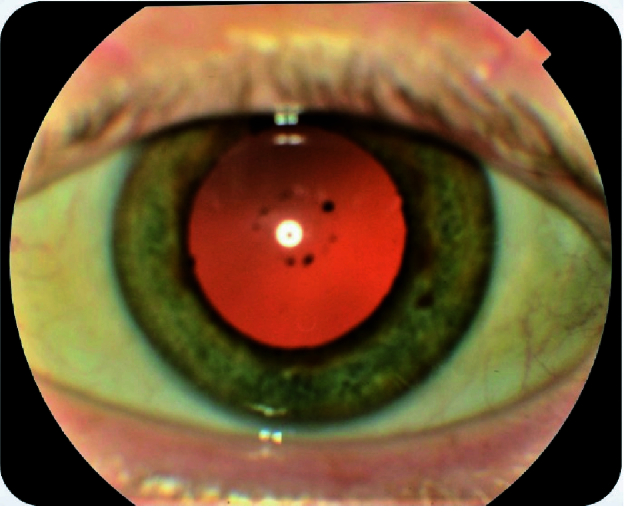
Anterior segment image of the right eye demonstrating the pigment deposits on anterior lens capsule. The left eye demonstrated similar pigment deposition.

**Figure 2:**
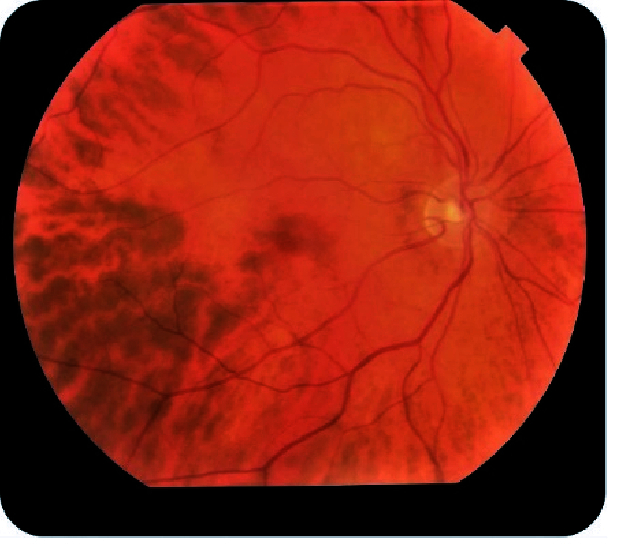
Fundus imaging of the right eye revealing a sunset glow-like appearance due to partial depigmentation of retinal pigment epithelium. The left eye appeared similar.

A 51-year-old Caucasian male with acral malignant melanoma initiated TIL for metastatic disease nine years following right toe amputation. Three months after treatment initiation, anterior segment exam of both eyes revealed diffuse pigment dusting on the corneal endothelium, along with pigment deposits on the lens capsule and floating pigments in the anterior chamber [Figure 1]. Fundus photography of both eyes revealed partial depigmentation of the retinal pigment epithelium (RPE), similar to the sunset glow appearance in Vogt-Koyanagi-Harada (VKH) disease [Figure 2].

Optical coherence tomography (OCT) demonstrated normal integrity of outer retinal structures explaining preserved visual acuity. Full field electroretinography (ERG) did not demonstrate rod or cone dysfunction at any voltage, supporting the fact that changes occurred only in RPE without visual sequelae. The patient was subsequently removed from the trial. Twenty-two months later, the patient was asymptomatic.

##  DISCUSSION 

This case report highlights ocular side effects associated with TIL use in the treatment of acral malignant melanoma. Symptoms often parallel those of VKH disease, which demonstrates a heightened immune response to tyrosinase ${\;}_{450-462}$ and gp100 ${\;}_{44-59}$ , two melanocyte antigens found in the ears, skin, hair, meninges, and eyes.^[4]^ Our patient did not demonstrate dermatologic manifestations such as vitiligo and poliosis in contrast to Yeh et al's 2009 case.^[4]^ He was asymptomatic with ERG records within normal limits.

The early ocular presentation of VKH is characterized by diffuse choroiditis, exudative retinal detachment, and hyperemia of the optic discs.^[5]^ The late phase demonstrates clumping and migration of RPE with fundal depigmentation.^[5]^ Further studies on patients who receive TIL will help us better understand the disease entity, while allowing us to better understand the pathophysiology of similar autoimmune disorders such as VKH, potentially helping to find an immune target treatment.

##  Financial Support and Sponsorship

This work was supported in part by an unrestricted institutional grant from Research to Prevent Blindness, NY, NY.

##  Conflicts of Interest

None.
